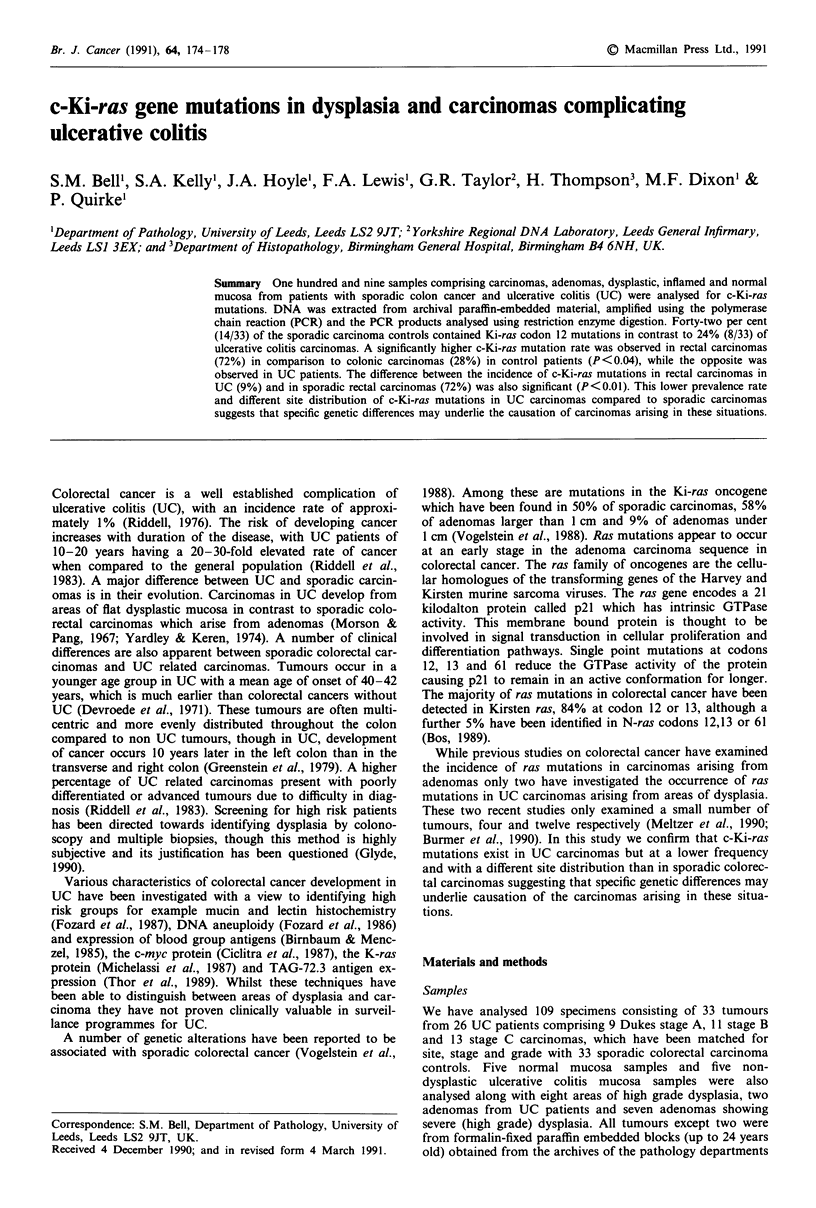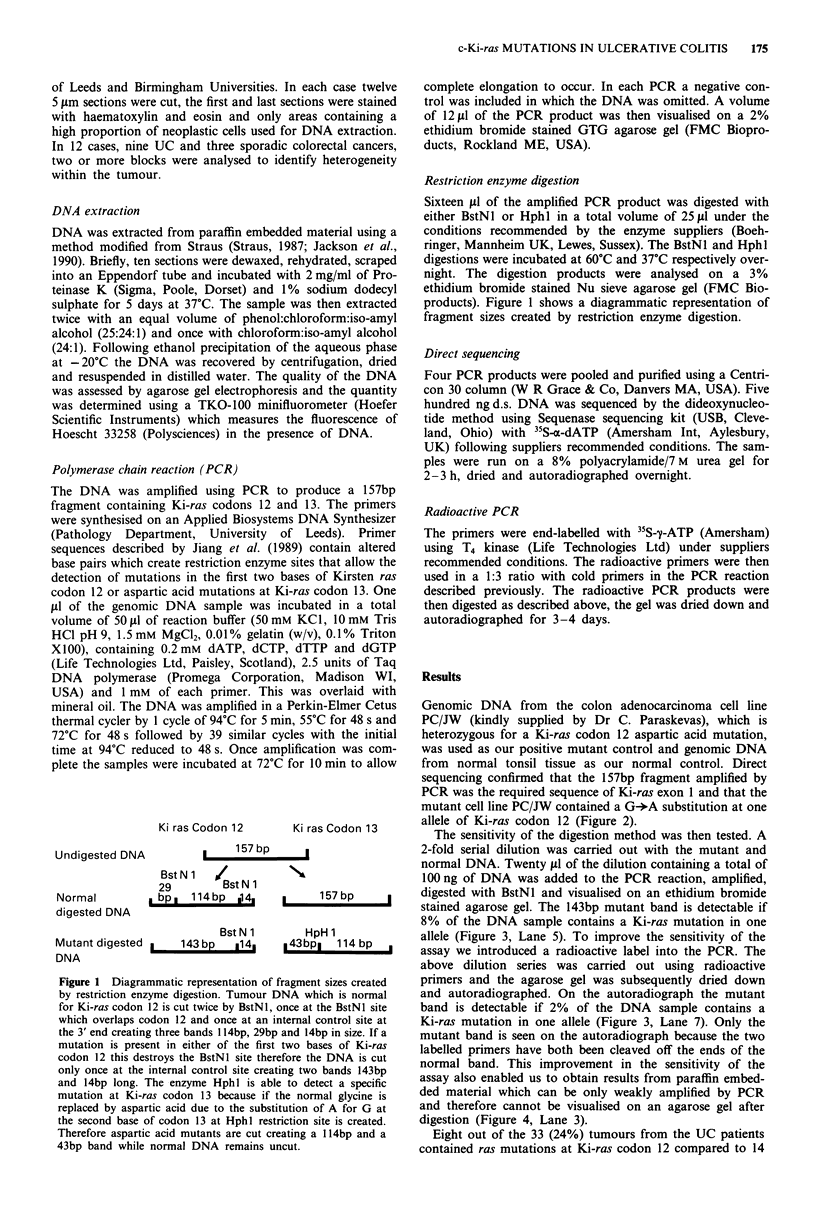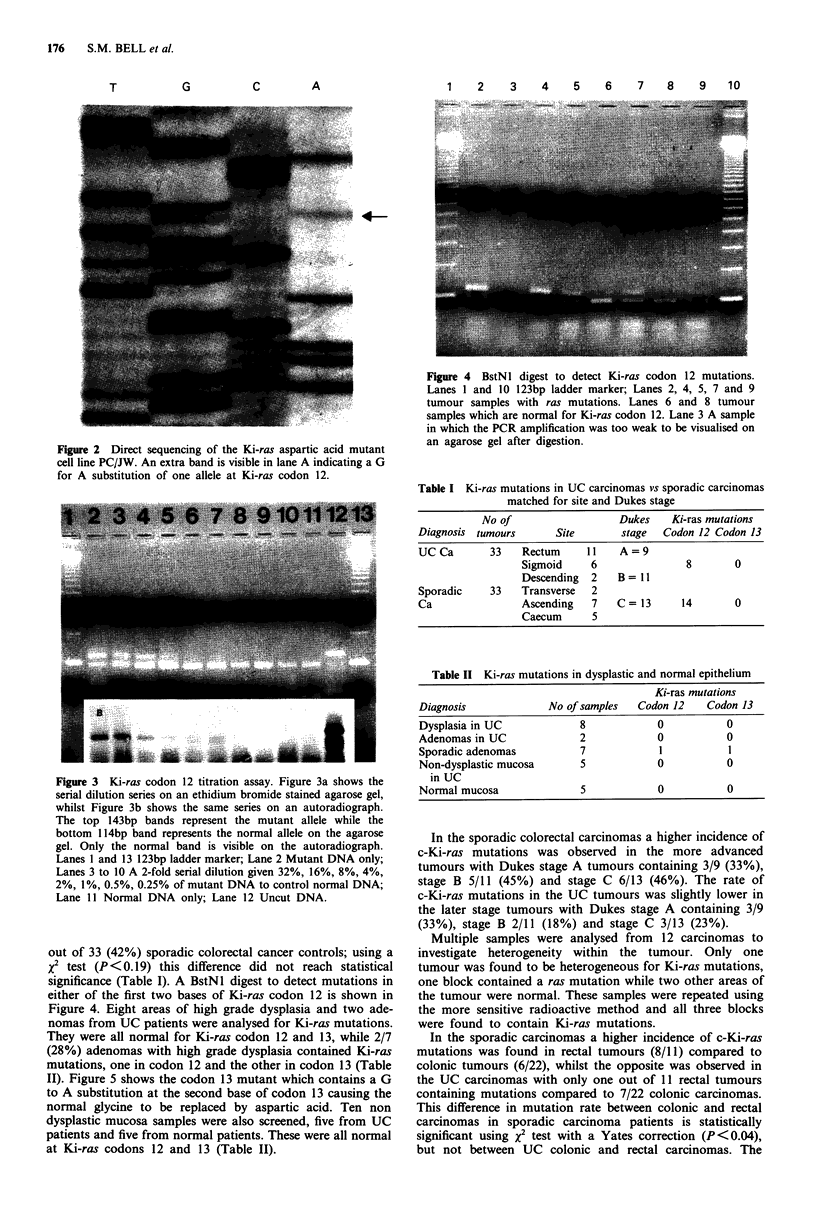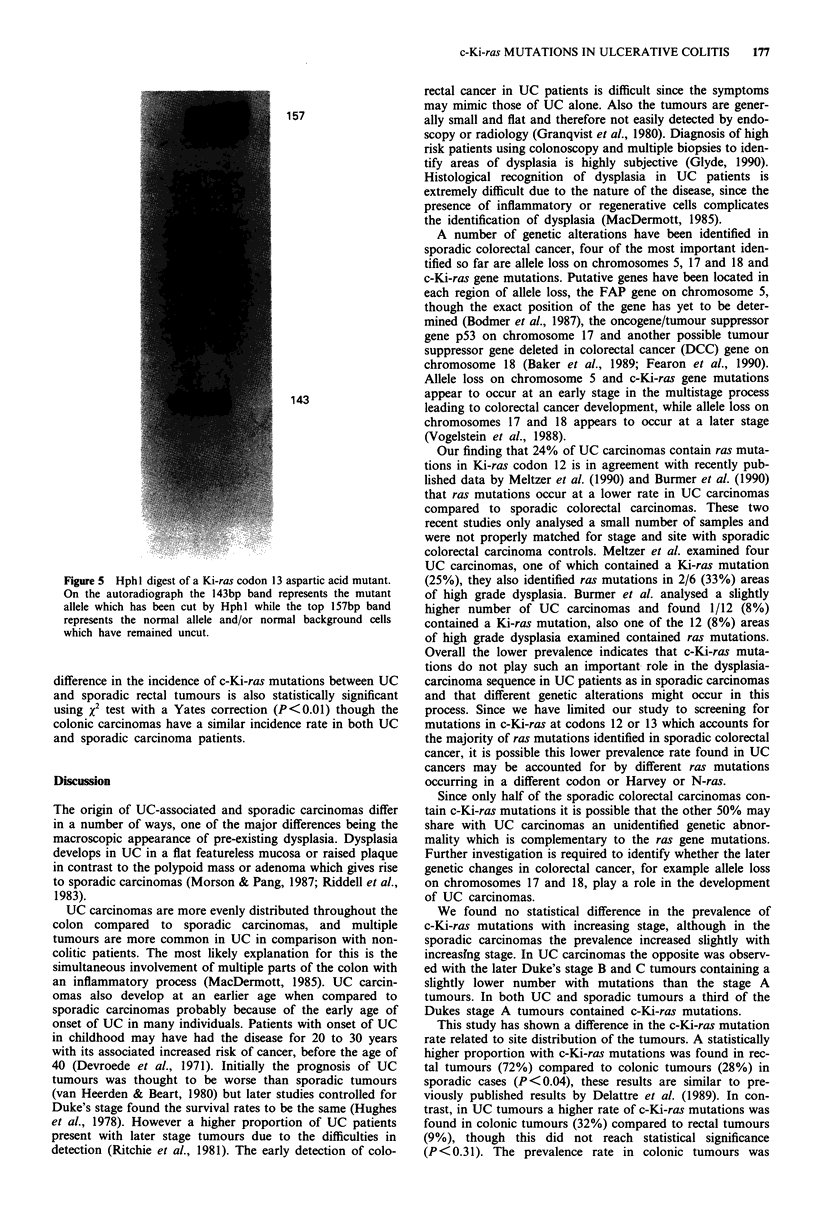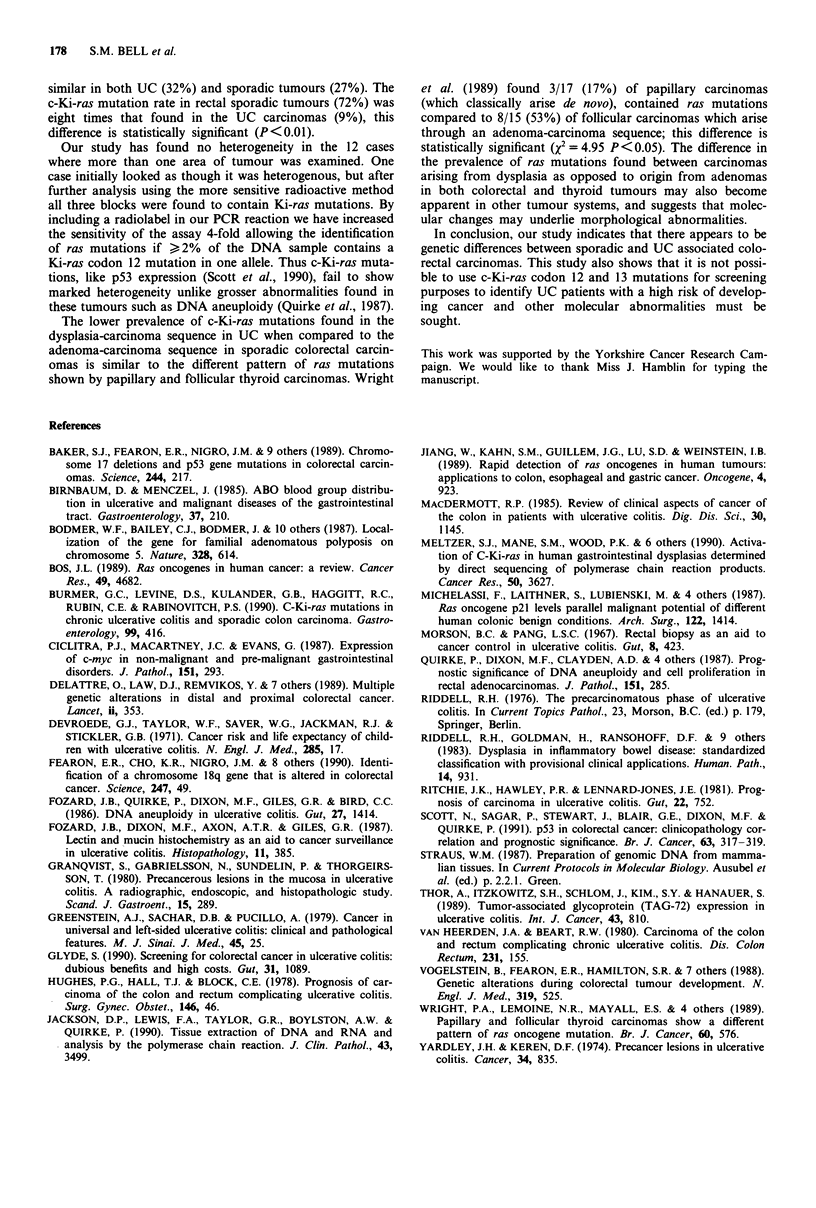# c-Ki-ras gene mutations in dysplasia and carcinomas complicating ulcerative colitis.

**DOI:** 10.1038/bjc.1991.264

**Published:** 1991-07

**Authors:** S. M. Bell, S. A. Kelly, J. A. Hoyle, F. A. Lewis, G. R. Taylor, H. Thompson, M. F. Dixon, P. Quirke

**Affiliations:** Department of Pathology, University of Leeds, UK.

## Abstract

**Images:**


Br. J. Cancer (1991), 64, 174-178 © Macmillan Press Ltd., 1991

### c-Ki-*ras* gene mutations in dysplasia and carcinomas complicating ulcerative colitis

S.M. Bell[1], S.A. Kelly[1], J.A. Hoyle[1], F.A. Lewis[1], G.R. Taylor[2], H. Thompson[3], M.F. Dixon[1] & P. Quirke[1]

[1]*Department of Pathology, University of Leeds, Leeds LS2 9JT;* [2]*Yorkshire Regional DNA Laboratory, Leeds General Infirmary, Leeds LS1 3EX; and* [3]*Department of Histopathology, Birmingham General Hospital, Birmingham B4 6NH, UK.*

**Summary** One hundred and nine samples comprising carcinomas, adenomas, dysplastic, inflamed and normal mucosa from patients with sporadic colon cancer and ulcerative colitis (UC) were analysed for c-Ki-*ras* mutations. DNA was extracted from archival paraffin-embedded material, amplified using the polymerase chain reaction (PCR) and the PCR products analysed using restriction enzyme digestion. Forty-two per cent (14/33) of the sporadic carcinoma controls contained Ki-*ras* codon 12 mutations in contrast to 24% (8/33) of ulcerative colitis carcinomas. A significantly higher c-Ki-*ras* mutation rate was observed in rectal carcinomas (72%) in comparison to colonic carcinomas (28%) in control patients ($P<0.04$), while the opposite was observed in UC patients. The difference between the incidence of c-Ki-*ras* mutations in rectal carcinomas in UC (9%) and in sporadic rectal carcinomas (72%) was also significant ($P<0.01$). This lower prevalence rate and different site distribution of c-Ki-*ras* mutations in UC carcinomas compared to sporadic carcinomas suggests that specific genetic differences may underlie the causation of carcinomas arising in these situations.

Colorectal cancer is a well established complication of ulcerative colitis (UC), with an incidence rate of approximately 1% (Riddell, 1976). The risk of developing cancer increases with duration of the disease, with UC patients of 10–20 years having a 20–30-fold elevated rate of cancer when compared to the general population (Riddell *et al.*, 1983). A major difference between UC and sporadic carcinomas is in their evolution. Carcinomas in UC develop from areas of flat dysplastic mucosa in contrast to sporadic colorectal carcinomas which arise from adenomas (Morson & Pang, 1967; Yardley & Keren, 1974). A number of clinical differences are also apparent between sporadic colorectal carcinomas and UC related carcinomas. Tumours occur in a younger age group in UC with a mean age of onset of 40–42 years, which is much earlier than colorectal cancers without UC (Devroede *et al.*, 1971). These tumours are often multicentric and more evenly distributed throughout the colon compared to non UC tumours, though in UC, development of cancer occurs 10 years later in the left colon than in the transverse and right colon (Greenstein *et al.*, 1979). A higher percentage of UC related carcinomas present with poorly differentiated or advanced tumours due to difficulty in diagnosis (Riddell *et al.*, 1983). Screening for high risk patients has been directed towards identifying dysplasia by colonoscopy and multiple biopsies, though this method is highly subjective and its justification has been questioned (Glyde, 1990).

Various characteristics of colorectal cancer development in UC have been investigated with a view to identifying high risk groups for example mucin and lectin histochemistry (Fozard *et al.*, 1987), DNA aneuploidy (Fozard *et al.*, 1986) and expression of blood group antigens (Birnbaum & Menczel, 1985), the c-*myc* protein (Ciclitra *et al.*, 1987), the K-*ras* protein (Michelassi *et al.*, 1987) and TAG-72.3 antigen expression (Thor *et al.*, 1989). Whilst these techniques have been able to distinguish between areas of dysplasia and carcinoma they have not proven clinically valuable in surveillance programmes for UC.

A number of genetic alterations have been reported to be associated with sporadic colorectal cancer (Vogelstein *et al.*, 1988). Among these are mutations in the Ki-*ras* oncogene which have been found in 50% of sporadic carcinomas, 58% of adenomas larger than 1 cm and 9% of adenomas under 1 cm (Vogelstein *et al.*, 1988). *Ras* mutations appear to occur at an early stage in the adenoma carcinoma sequence in colorectal cancer. The *ras* family of oncogenes are the cellular homologues of the transforming genes of the Harvey and Kirsten murine sarcoma viruses. The *ras* gene encodes a 21 kilodalton protein called p21 which has intrinsic GTPase activity. This membrane bound protein is thought to be involved in signal transduction in cellular proliferation and differentiation pathways. Single point mutations at codons 12, 13 and 61 reduce the GTPase activity of the protein causing p21 to remain in an active conformation for longer. The majority of *ras* mutations in colorectal cancer have been detected in Kirsten *ras*, 84% at codon 12 or 13, although a further 5% have been identified in N-*ras* codons 12,13 or 61 (Bos, 1989).

While previous studies on colorectal cancer have examined the incidence of *ras* mutations in carcinomas arising from adenomas only two have investigated the occurrence of *ras* mutations in UC carcinomas arising from areas of dysplasia. These two recent studies only examined a small number of tumours, four and twelve respectively (Meltzer *et al.*, 1990; Burmer *et al.*, 1990). In this study we confirm that c-Ki-*ras* mutations exist in UC carcinomas but at a lower frequency and with a different site distribution than in sporadic colorectal carcinomas suggesting that specific genetic differences may underlie causation of the carcinomas arising in these situations.

### Materials and methods

### *Samples*

We have analysed 109 specimens consisting of 33 tumours from 26 UC patients comprising 9 Dukes stage A, 11 stage B and 13 stage C carcinomas, which have been matched for site, stage and grade with 33 sporadic colorectal carcinoma controls. Five normal mucosa samples and five non-dysplastic ulcerative colitis mucosa samples were also analysed along with eight areas of high grade dysplasia, two adenomas from UC patients and seven adenomas showing severe (high grade) dysplasia. All tumours except two were from formalin-fixed paraffin embedded blocks (up to 24 years old) obtained from the archives of the pathology departments

Correspondence: S.M. Bell, Department of Pathology, University of Leeds, Leeds LS2 9JT, UK.
Received 4 December 1990; and in revised form 4 March 1991.

of Leeds and Birmingham Universities. In each case twelve 5 μm sections were cut, the first and last sections were stained with haematoxylin and eosin and only areas containing a high proportion of neoplastic cells used for DNA extraction. In 12 cases, nine UC and three sporadic colorectal cancers, two or more blocks were analysed to identify heterogeneity within the tumour.

### *DNA extraction*

DNA was extracted from paraffin embedded material using a method modified from Straus (Straus, 1987; Jackson *et al.*, 1990). Briefly, ten sections were dewaxed, rehydrated, scraped into an Eppendorf tube and incubated with 2 mg/ml of Proteinase K (Sigma, Poole, Dorset) and 1% sodium dodecyl sulphate for 5 days at 37°C. The sample was then extracted twice with an equal volume of phenol:chloroform:iso-amyl alcohol (25:24:1) and once with chloroform:iso-amyl alcohol (24:1). Following ethanol precipitation of the aqueous phase at −20°C the DNA was recovered by centrifugation, dried and resuspended in distilled water. The quality of the DNA was assessed by agarose gel electrophoresis and the quantity was determined using a TKO-100 minifluorometer (Hoefer Scientific Instruments) which measures the fluorescence of Hoescht 33258 (Polysciences) in the presence of DNA.

### *Polymerase chain reaction (PCR)*

The DNA was amplified using PCR to produce a 157bp fragment containing Ki-*ras* codons 12 and 13. The primers were synthesised on an Applied Biosystems DNA Synthesizer (Pathology Department, University of Leeds). Primer sequences described by Jiang *et al.* (1989) contain altered base pairs which create restriction enzyme sites that allow the detection of mutations in the first two bases of Kirsten *ras* codon 12 or aspartic acid mutations at Ki-*ras* codon 13. One μl of the genomic DNA sample was incubated in a total volume of 50 μl of reaction buffer (50 mM KCl, 10 mM Tris HCl pH 9, 1.5 mM $MgCl_2$, 0.01% gelatin (w/v), 0.1% Triton X100), containing 0.2 mM dATP, dCTP, dTTP and dGTP (Life Technologies Ltd, Paisley, Scotland), 2.5 units of Taq DNA polymerase (Promega Corporation, Madison WI, USA) and 1 mM of each primer. This was overlaid with mineral oil. The DNA was amplified in a Perkin-Elmer Cetus thermal cycler by 1 cycle of 94°C for 5 min, 55°C for 48 s and 72°C for 48 s followed by 39 similar cycles with the initial time at 94°C reduced to 48 s. Once amplification was complete the samples were incubated at 72°C for 10 min to allow

**Figure 1** Diagrammatic representation of fragment sizes created by restriction enzyme digestion. Tumour DNA which is normal for Ki-*ras* codon 12 is cut twice by BstN1, once at the BstN1 site which overlaps codon 12 and once at an internal control site at the 3′ end creating three bands 114bp, 29bp and 14bp in size. If a mutation is present in either of the first two bases of Ki-*ras* codon 12 this destroys the BstN1 site therefore the DNA is cut only once at the internal control site creating two bands 143bp and 14bp long. The enzyme Hph1 is able to detect a specific mutation at Ki-*ras* codon 13 because if the normal glycine is replaced by aspartic acid due to the substitution of A for G at the second base of codon 13 at Hph1 restriction site is created. Therefore aspartic acid mutants are cut creating a 114bp and a 43bp band while normal DNA remains uncut.

complete elongation to occur. In each PCR a negative control was included in which the DNA was omitted. A volume of 12 μl of the PCR product was then visualised on a 2% ethidium bromide stained GTG agarose gel (FMC Bioproducts, Rockland ME, USA).

### *Restriction enzyme digestion*

Sixteen μl of the amplified PCR product was digested with either BstN1 or Hph1 in a total volume of 25 μl under the conditions recommended by the enzyme suppliers (Boehringer, Mannheim UK, Lewes, Sussex). The BstN1 and Hph1 digestions were incubated at 60°C and 37°C respectively overnight. The digestion products were analysed on a 3% ethidium bromide stained Nu sieve agarose gel (FMC Bioproducts). Figure 1 shows a diagrammatic representation of fragment sizes created by restriction enzyme digestion.

### *Direct sequencing*

Four PCR products were pooled and purified using a Centricon 30 column (W R Grace & Co, Danvers MA, USA). Five hundred ng d.s. DNA was sequenced by the dideoxynucleotide method using Sequenase sequencing kit (USB, Cleveland, Ohio) with $^{35}S$-α-dATP (Amersham Int, Aylesbury, UK) following suppliers recommended conditions. The samples were run on a 8% polyacrylamide/7 M urea gel for 2–3 h, dried and autoradiographed overnight.

### *Radioactive PCR*

The primers were end-labelled with $^{35}S$-γ-ATP (Amersham) using $T_4$ kinase (Life Technologies Ltd) under suppliers recommended conditions. The radioactive primers were then used in a 1:3 ratio with cold primers in the PCR reaction described previously. The radioactive PCR products were then digested as described above, the gel was dried down and autoradiographed for 3–4 days.

### Results

Genomic DNA from the colon adenocarcinoma cell line PC/JW (kindly supplied by Dr C. Paraskevas), which is heterozygous for a Ki-*ras* codon 12 aspartic acid mutation, was used as our positive mutant control and genomic DNA from normal tonsil tissue as our normal control. Direct sequencing confirmed that the 157bp fragment amplified by PCR was the required sequence of Ki-*ras* exon 1 and that the mutant cell line PC/JW contained a G→A substitution at one allele of Ki-*ras* codon 12 (Figure 2).

The sensitivity of the digestion method was then tested. A 2-fold serial dilution was carried out with the mutant and normal DNA. Twenty μl of the dilution containing a total of 100 ng of DNA was added to the PCR reaction, amplified, digested with BstN1 and visualised on an ethidium bromide stained agarose gel. The 143bp mutant band is detectable if 8% of the DNA sample contains a Ki-*ras* mutation in one allele (Figure 3, Lane 5). To improve the sensitivity of the assay we introduced a radioactive label into the PCR. The above dilution series was carried out using radioactive primers and the agarose gel was subsequently dried down and autoradiographed. On the autoradiograph the mutant band is detectable if 2% of the DNA sample contains a Ki-*ras* mutation in one allele (Figure 3, Lane 7). Only the mutant band is seen on the autoradiograph because the two labelled primers have both been cleaved off the ends of the normal band. This improvement in the sensitivity of the assay also enabled us to obtain results from paraffin embedded material which can be only weakly amplified by PCR and therefore cannot be visualised on an agarose gel after digestion (Figure 4, Lane 3).

Eight out of the 33 (24%) tumours from the UC patients contained *ras* mutations at Ki-*ras* codon 12 compared to 14

**Figure 2** Direct sequencing of the Ki-*ras* aspartic acid mutant cell line PC/JW. An extra band is visible in lane A indicating a G for A substitution of one allele at Ki-*ras* codon 12.

**Figure 3** Ki-*ras* codon 12 titration assay. Figure 3a shows the serial dilution series on an ethidium bromide stained agarose gel, whilst Figure 3b shows the same series on an autoradiograph. The top 143bp bands represent the mutant allele while the bottom 114bp band represents the normal allele on the agarose gel. Only the normal band is visible on the autoradiograph. Lanes 1 and 13 123bp ladder marker; Lane 2 Mutant DNA only; Lanes 3 to 10 A 2-fold serial dilution given 32%, 16%, 8%, 4%, 2%, 1%, 0.5%, 0.25% of mutant DNA to control normal DNA; Lane 11 Normal DNA only; Lane 12 Uncut DNA.

**Figure 4** BstN1 digest to detect Ki-*ras* codon 12 mutations. Lanes 1 and 10 123bp ladder marker; Lanes 2, 4, 5, 7 and 9 tumour samples with *ras* mutations. Lanes 6 and 8 tumour samples which are normal for Ki-*ras* codon 12. Lane 3 A sample in which the PCR amplification was too weak to be visualised on an agarose gel after digestion.

**Table I** Ki-*ras* mutations in UC carcinomas *vs* sporadic carcinomas matched for site and Dukes stage

| *Diagnosis* | *No of tumours* | *Site* | | *Dukes stage* | *Ki-*ras *mutations* Codon 12 | *Codon 13* |
|---|---|---|---|---|---|---|
| UC Ca | 33 | Rectum | 11 | A = 9 | | |
| | | Sigmoid | 6 | | 8 | 0 |
| | | Descending | 2 | B = 11 | | |
| Sporadic | 33 | Transverse | 2 | | | |
| Ca | | Ascending | 7 | C = 13 | 14 | 0 |
| | | Caecum | 5 | | | |

**Table II** Ki-*ras* mutations in dysplastic and normal epithelium

| *Diagnosis* | *No of samples* | *Ki-*ras *mutations* Codon 12 | *Codon 13* |
|---|---|---|---|
| Dysplasia in UC | 8 | 0 | 0 |
| Adenomas in UC | 2 | 0 | 0 |
| Sporadic adenomas | 7 | 1 | 1 |
| Non-dysplastic mucosa in UC | 5 | 0 | 0 |
| Normal mucosa | 5 | 0 | 0 |

out of 33 (42%) sporadic colorectal cancer controls; using a $\chi^2$ test ($P<0.19$) this difference did not reach statistical significance (Table I). A BstN1 digest to detect mutations in either of the first two bases of Ki-*ras* codon 12 is shown in Figure 4. Eight areas of high grade dysplasia and two adenomas from UC patients were analysed for Ki-*ras* mutations. They were all normal for Ki-*ras* codon 12 and 13, while 2/7 (28%) adenomas with high grade dysplasia contained Ki-*ras* mutations, one in codon 12 and the other in codon 13 (Table II). Figure 5 shows the codon 13 mutant which contains a G to A substitution at the second base of codon 13 causing the normal glycine to be replaced by aspartic acid. Ten non dysplastic mucosa samples were also screened, five from UC patients and five from normal patients. These were all normal at Ki-*ras* codons 12 and 13 (Table II).

In the sporadic colorectal carcinomas a higher incidence of c-Ki-*ras* mutations was observed in the more advanced tumours with Dukes stage A tumours containing 3/9 (33%), stage B 5/11 (45%) and stage C 6/13 (46%). The rate of c-Ki-*ras* mutations in the UC tumours was slightly lower in the later stage tumours with Dukes stage A containing 3/9 (33%), stage B 2/11 (18%) and stage C 3/13 (23%).

Multiple samples were analysed from 12 carcinomas to investigate heterogeneity within the tumour. Only one tumour was found to be heterogeneous for Ki-*ras* mutations, one block contained a *ras* mutation while two other areas of the tumour were normal. These samples were repeated using the more sensitive radioactive method and all three blocks were found to contain Ki-*ras* mutations.

In the sporadic carcinomas a higher incidence of c-Ki-*ras* mutations was found in rectal tumours (8/11) compared to colonic tumours (6/22), whilst the opposite was observed in the UC carcinomas with only one out of 11 rectal tumours containing mutations compared to 7/22 colonic carcinomas. This difference in mutation rate between colonic and rectal carcinomas in sporadic carcinoma patients is statistically significant using $\chi^2$ test with a Yates correction ($P<0.04$), but not between UC colonic and rectal carcinomas. The

**Figure 5** Hph1 digest of a Ki-*ras* codon 13 aspartic acid mutant. On the autoradiograph the 143bp band represents the mutant allele which has been cut by Hph1 while the top 157bp band represents the normal allele and/or normal background cells which have remained uncut.

difference in the incidence of c-Ki-*ras* mutations between UC and sporadic rectal tumours is also statistically significant using $\chi^2$ test with a Yates correction ($P<0.01$) though the colonic carcinomas have a similar incidence rate in both UC and sporadic carcinoma patients.

### Discussion

The origin of UC-associated and sporadic carcinomas differ in a number of ways, one of the major differences being the macroscopic appearance of pre-existing dysplasia. Dysplasia develops in UC in a flat featureless mucosa or raised plaque in contrast to the polypoid mass or adenoma which gives rise to sporadic carcinomas (Morson & Pang, 1987; Riddell *et al.*, 1983).

UC carcinomas are more evenly distributed throughout the colon compared to sporadic carcinomas, and multiple tumours are more common in UC in comparison with non-colitic patients. The most likely explanation for this is the simultaneous involvement of multiple parts of the colon with an inflammatory process (MacDermott, 1985). UC carcinomas also develop at an earlier age when compared to sporadic carcinomas probably because of the early age of onset of UC in many individuals. Patients with onset of UC in childhood may have had the disease for 20 to 30 years with its associated increased risk of cancer, before the age of 40 (Devroede *et al.*, 1971). Initially the prognosis of UC tumours was thought to be worse than sporadic tumours (van Heerden & Beart, 1980) but later studies controlled for Duke's stage found the survival rates to be the same (Hughes *et al.*, 1978). However a higher proportion of UC patients present with later stage tumours due to the difficulties in detection (Ritchie *et al.*, 1981). The early detection of colorectal cancer in UC patients is difficult since the symptoms may mimic those of UC alone. Also the tumours are generally small and flat and therefore not easily detected by endoscopy or radiology (Granqvist *et al.*, 1980). Diagnosis of high risk patients using colonoscopy and multiple biopsies to identify areas of dysplasia is highly subjective (Glyde, 1990). Histological recognition of dysplasia in UC patients is extremely difficult due to the nature of the disease, since the presence of inflammatory or regenerative cells complicates the identification of dysplasia (MacDermott, 1985).

A number of genetic alterations have been identified in sporadic colorectal cancer, four of the most important identified so far are allele loss on chromosomes 5, 17 and 18 and c-Ki-*ras* gene mutations. Putative genes have been located in each region of allele loss, the FAP gene on chromosome 5, though the exact position of the gene has yet to be determined (Bodmer *et al.*, 1987), the oncogene/tumour suppressor gene p53 on chromosome 17 and another possible tumour suppressor gene deleted in colorectal cancer (DCC) gene on chromosome 18 (Baker *et al.*, 1989; Fearon *et al.*, 1990). Allele loss on chromosome 5 and c-Ki-*ras* gene mutations appear to occur at an early stage in the multistage process leading to colorectal cancer development, while allele loss on chromosomes 17 and 18 appears to occur at a later stage (Vogelstein *et al.*, 1988).

Our finding that 24% of UC carcinomas contain *ras* mutations in Ki-*ras* codon 12 is in agreement with recently published data by Meltzer *et al.* (1990) and Burmer *et al.* (1990) that *ras* mutations occur at a lower rate in UC carcinomas compared to sporadic colorectal carcinomas. These two recent studies only analysed a small number of samples and were not properly matched for stage and site with sporadic colorectal carcinoma controls. Meltzer *et al.* examined four UC carcinomas, one of which contained a Ki-*ras* mutation (25%), they also identified *ras* mutations in 2/6 (33%) areas of high grade dysplasia. Burmer *et al.* analysed a slightly higher number of UC carcinomas and found 1/12 (8%) contained a Ki-*ras* mutation, also one of the 12 (8%) areas of high grade dysplasia examined contained *ras* mutations. Overall the lower prevalence indicates that c-Ki-*ras* mutations do not play such an important role in the dysplasia-carcinoma sequence in UC patients as in sporadic carcinomas and that different genetic alterations might occur in this process. Since we have limited our study to screening for mutations in c-Ki-*ras* at codons 12 or 13 which accounts for the majority of *ras* mutations identified in sporadic colorectal cancer, it is possible this lower prevalence rate found in UC cancers may be accounted for by different *ras* mutations occurring in a different codon or Harvey or N-*ras*.

Since only half of the sporadic colorectal carcinomas contain c-Ki-*ras* mutations it is possible that the other 50% may share with UC carcinomas an unidentified genetic abnormality which is complementary to the *ras* gene mutations. Further investigation is required to identify whether the later genetic changes in colorectal cancer, for example allele loss on chromosomes 17 and 18, play a role in the development of UC carcinomas.

We found no statistical difference in the prevalence of c-Ki-*ras* mutations with increasing stage, although in the sporadic carcinomas the prevalence increased slightly with increasing stage. In UC carcinomas the opposite was observed with the later Duke's stage B and C tumours containing a slightly lower number with mutations than the stage A tumours. In both UC and sporadic tumours a third of the Dukes stage A tumours contained c-Ki-*ras* mutations.

This study has shown a difference in the c-Ki-*ras* mutation rate related to site distribution of the tumours. A statistically higher proportion with c-Ki-*ras* mutations was found in rectal tumours (72%) compared to colonic tumours (28%) in sporadic cases ($P<0.04$), these results are similar to previously published results by Delattre *et al.* (1989). In contrast, in UC tumours a higher rate of c-Ki-*ras* mutations was found in colonic tumours (32%) compared to rectal tumours (9%), though this did not reach statistical significance ($P<0.31$). The prevalence rate in colonic tumours was similar in both UC (32%) and sporadic tumours (27%). The c-Ki-*ras* mutation rate in rectal sporadic tumours (72%) was eight times that found in the UC carcinomas (9%), this difference is statistically significant ($P < 0.01$).

Our study has found no heterogeneity in the 12 cases where more than one area of tumour was examined. One case initially looked as though it was heterogenous, but after further analysis using the more sensitive radioactive method all three blocks were found to contain Ki-*ras* mutations. By including a radiolabel in our PCR reaction we have increased the sensitivity of the assay 4-fold allowing the identification of *ras* mutations if $\geqslant 2\%$ of the DNA sample contains a Ki-*ras* codon 12 mutation in one allele. Thus c-Ki-*ras* mutations, like p53 expression (Scott *et al.*, 1990), fail to show marked heterogeneity unlike grosser abnormalities found in these tumours such as DNA aneuploidy (Quirke *et al.*, 1987).

The lower prevalence of c-Ki-*ras* mutations found in the dysplasia-carcinoma sequence in UC when compared to the adenoma-carcinoma sequence in sporadic colorectal carcinomas is similar to the different pattern of *ras* mutations shown by papillary and follicular thyroid carcinomas. Wright *et al.* (1989) found 3/17 (17%) of papillary carcinomas (which classically arise *de novo*), contained *ras* mutations compared to 8/15 (53%) of follicular carcinomas which arise through an adenoma-carcinoma sequence; this difference is statistically significant ($\chi^2 = 4.95$ $P < 0.05$). The difference in the prevalence of *ras* mutations found between carcinomas arising from dysplasia as opposed to origin from adenomas in both colorectal and thyroid tumours may also become apparent in other tumour systems, and suggests that molecular changes may underlie morphological abnormalities.

In conclusion, our study indicates that there appears to be genetic differences between sporadic and UC associated colorectal carcinomas. This study also shows that it is not possible to use c-Ki-*ras* codon 12 and 13 mutations for screening purposes to identify UC patients with a high risk of developing cancer and other molecular abnormalities must be sought.

This work was supported by the Yorkshire Cancer Research Campaign. We would like to thank Miss J. Hamblin for typing the manuscript.

### References

BAKER, S.J., FEARON, E.R., NIGRO, J.M. & 9 others (1989). Chromosome 17 deletions and p53 gene mutations in colorectal carcinomas. *Science*, **244**, 217.

BIRNBAUM, D. & MENCZEL, J. (1985). ABO blood group distribution in ulcerative and malignant diseases of the gastrointestinal tract. *Gastroenterology*, **37**, 210.

BODMER, W.F., BAILEY, C.J., BODMER, J. & 10 others (1987). Localization of the gene for familial adenomatous polyposis on chromosome 5. *Nature*, **328**, 614.

BOS, J.L. (1989). *Ras* oncogenes in human cancer: a review. *Cancer Res.*, **49**, 4682.

BURMER, G.C., LEVINE, D.S., KULANDER, G.B., HAGGITT, R.C., RUBIN, C.E. & RABINOVITCH, P.S. (1990). C-Ki-*ras* mutations in chronic ulcerative colitis and sporadic colon carcinoma. *Gastroenterology*, **99**, 416.

CICLITRA, P.J., MACARTNEY, J.C. & EVANS, G. (1987). Expression of c-*myc* in non-malignant and pre-malignant gastrointestinal disorders. *J. Pathol.*, **151**, 293.

DELATTRE, O., LAW, D.J., REMVIKOS, Y. & 7 others (1989). Multiple genetic alterations in distal and proximal colorectal cancer. *Lancet*, **ii**, 353.

DEVROEDE, G.J., TAYLOR, W.F., SAVER, W.G., JACKMAN, R.J. & STICKLER, G.B. (1971). Cancer risk and life expectancy of children with ulcerative colitis. *N. Engl. J. Med.*, **285**, 17.

FEARON, E.R., CHO, K.R., NIGRO, J.M. & 8 others (1990). Identification of a chromosome 18q gene that is altered in colorectal cancer. *Science*, **247**, 49.

FOZARD, J.B., QUIRKE, P., DIXON, M.F., GILES, G.R. & BIRD, C.C. (1986). DNA aneuploidy in ulcerative colitis. *Gut*, **27**, 1414.

FOZARD, J.B., DIXON, M.F., AXON, A.T.R. & GILES, G.R. (1987). Lectin and mucin histochemistry as an aid to cancer surveillance in ulcerative colitis. *Histopathology*, **11**, 385.

GRANQVIST, S., GABRIELSSON, N., SUNDELIN, P. & THORGEIRSSON, T. (1980). Precancerous lesions in the mucosa in ulcerative colitis. A radiographic, endoscopic, and histopathologic study. *Scand. J. Gastroent.*, **15**, 289.

GREENSTEIN, A.J., SACHAR, D.B. & PUCILLO, A. (1979). Cancer in universal and left-sided ulcerative colitis: clinical and pathological features. *M. J. Sinai. J. Med.*, **45**, 25.

GLYDE, S. (1990). Screening for colorectal cancer in ulcerative colitis: dubious benefits and high costs. *Gut*, **31**, 1089.

HUGHES, P.G., HALL, T.J. & BLOCK, C.E. (1978). Prognosis of carcinoma of the colon and rectum complicating ulcerative colitis. *Surg. Gynec. Obstet.*, **146**, 46.

JACKSON, D.P., LEWIS, F.A., TAYLOR, G.R., BOYLSTON, A.W. & QUIRKE, P. (1990). Tissue extraction of DNA and RNA and analysis by the polymerase chain reaction. *J. Clin. Pathol.*, **43**, 3499.

JIANG, W., KAHN, S.M., GUILLEM, J.G., LU, S.D. & WEINSTEIN, I.B. (1989). Rapid detection of *ras* oncogenes in human tumours: applications to colon, esophageal and gastric cancer. *Oncogene*, **4**, 923.

MACDERMOTT, R.P. (1985). Review of clinical aspects of cancer of the colon in patients with ulcerative colitis. *Dig. Dis. Sci.*, **30**, 1145.

MELTZER, S.J., MANE, S.M., WOOD, P.K. & 6 others (1990). Activation of C-Ki-*ras* in human gastrointestinal dysplasias determined by direct sequencing of polymerase chain reaction products. *Cancer Res.*, **50**, 3627.

MICHELASSI, F., LAITHNER, S., LUBIENSKI, M. & 4 others (1987). *Ras* oncogene p21 levels parallel malignant potential of different human colonic benign conditions. *Arch. Surg.*, **122**, 1414.

MORSON, B.C. & PANG, L.S.C. (1967). Rectal biopsy as an aid to cancer control in ulcerative colitis. *Gut*, **8**, 423.

QUIRKE, P., DIXON, M.F., CLAYDEN, A.D. & 4 others (1987). Prognostic significance of DNA aneuploidy and cell proliferation in rectal adenocarcinomas. *J. Pathol.*, **151**, 285.

RIDDELL, R.H. (1976). The precarcinomatous phase of ulcerative colitis. In *Current Topics Pathol.*, 23, Morson, B.C. (ed.) p. 179, Springer, Berlin.

RIDDELL, R.H., GOLDMAN, H., RANSOHOFF, D.F. & 9 others (1983). Dysplasia in inflammatory bowel disease: standardized classification with provisional clinical applications. *Human. Path.*, **14**, 931.

RITCHIE, J.K., HAWLEY, P.R. & LENNARD-JONES, J.E. (1981). Prognosis of carcinoma in ulcerative colitis. *Gut*, **22**, 752.

SCOTT, N., SAGAR, P., STEWART, J., BLAIR, G.E., DIXON, M.F. & QUIRKE, P. (1991). p53 in colorectal cancer: clinicopathology correlation and prognostic significance. *Br. J. Cancer*, **63**, 317–319.

STRAUS, W.M. (1987). Preparation of genomic DNA from mammalian tissues. In *Current Protocols in Molecular Biology*. Ausubel *et al.* (ed.) p. 2.2.1. Green.

THOR, A., ITZKOWITZ, S.H., SCHLOM, J., KIM., S.Y. & HANAUER, S. (1989). Tumor-associated glycoprotein (TAG-72) expression in ulcerative colitis. *Int. J. Cancer*, **43**, 810.

VAN HEERDEN, J.A. & BEART, R.W. (1980). Carcinoma of the colon and rectum complicating chronic ulcerative colitis. *Dis. Colon Rectum*, **231**, 155.

VOGELSTEIN, B., FEARON, E.R., HAMILTON, S.R. & 7 others (1988). Genetic alterations during colorectal tumour development. *N. Engl. J. Med.*, **319**, 525.

WRIGHT, P.A., LEMOINE, N.R., MAYALL, E.S. & 4 others (1989). Papillary and follicular thyroid carcinomas show a different pattern of *ras* oncogene mutation. *Br. J. Cancer*, **60**, 576.

YARDLEY, J.H. & KEREN, D.F. (1974). Precancer lesions in ulcerative colitis. *Cancer*, **34**, 835.